# Associations between Skin Autofluorescence Levels with Cardiovascular Risk and Diabetes Complications in Patients with Type 2 Diabetes

**DOI:** 10.3390/biomedicines12040890

**Published:** 2024-04-17

**Authors:** Delia Reurean-Pintilei, Anca Pantea Stoian, Teodor Salmen, Roxana-Adriana Stoica, Liliana Mititelu-Tartau, Sandra Lazăr, Bogdan Timar

**Affiliations:** 1 Doctoral School of Medicine and Pharmacy, “Victor Babes” University of Medicine and Pharmacy, 300041 Timisoara, Romania; drdeliapintilei@gmail.com; 2 Centre for Molecular Research in Nephrology and Vascular Disease, “Victor Babes” University of Medicine and Pharmacy, 300041 Timisoara, Romania; 3 Department of Diabetes, Nutrition and Metabolic Diseases, Consultmed Medical Centre, 700544 Iasi, Romania; 4 Diabetes, Nutrition and Metabolic Diseases Department, “Carol Davila” University of Medicine and Pharmacy, 050474 Bucharest, Romania; 5 Doctoral School of “Carol Davila”, University of Medicine and Pharmacy, 050474 Bucharest, Romania; 6 Department of Pharmacology, Faculty of Medicine, “Grigore T. Popa” University of Medicine and Pharmacy, 700115 Iasi, Romania; 7 First Department of Internal Medicine, “Victor Babes” University of Medicine and Pharmacy, 300041 Timisoara, Romania; 8 Department of Hematology, Emergency Municipal Hospital Timisoara, 300041 Timisoara, Romania; 9 Second Department of Internal Medicine, “Victor Babes” University of Medicine and Pharmacy, 300041 Timisoara, Romania; 10 Department of Diabetes, Nutrition and Metabolic Diseases, “Pius Brinzeu” Emergency Hospital, 300723 Timisoara, Romania

**Keywords:** Advanced Glycation End Products (AGEs), skin autofluorescence, type 2 diabetes mellitus (T2DM), cardiovascular risk

## Abstract

Advanced Glycation End Products (AGEs) contribute to the pathophysiology of type 2 diabetes mellitus (T2DM) and cardiovascular (CV) diseases (CVDs), making their non-invasive assessment through skin autofluorescence (SAF) increasingly important. This study aims to investigate the relationship between SAF levels, cardiovascular risk, and diabetic complications in T2DM patients. We conducted a single-center, cross-sectional study at Consultmed Hospital in Iasi, Romania, including 885 T2DM patients. The assessment of SAF levels was performed with the AGE Reader™, (Diagnoptics, Groningen, The Netherlands). CVD prevalence was 13.9%, and according to CV risk category distribution, 6.1% fell into the moderate-risk, 1.13% into the high-risk, and 92.77% into the very-high-risk category. The duration of DM averaged 9.0 ± 4.4 years and the mean HbA1c was 7.1% ± 1.3. After adjusting for age and eGFR, HbA1c values showed a correlation with SAF levels in the multivariate regression model, where a 1 SD increase in HbA1c was associated with a 0.105 SD increase in SAF levels (Nagelkerke R^2^ = 0.110; *p* < 0.001). For predicting very high risk with an SAF cut-off of 2.35, sensitivity was 67.7% and specificity was 56.2%, with an AUC of 0.634 (95% CI 0.560–0.709, *p* = 0.001). In T2DM, elevated SAF levels were associated with higher CV risk and HbA1c values, with 2.35 identified as the optimal SAF cut-off for very high CV risk.

## 1. Introduction

Advanced Glycation End Products (AGEs) are detrimental byproducts of hyperglycemia in diabetes mellitus (DM) and aging in the non-diabetic population, with their accumulation exacerbating glucose metabolism imbalances, metabolic syndrome (MetS), and chronic diseases, including cardiovascular (CV) disease, chronic kidney disease (CKD), and neurodegenerative disorders [1,2,3,4,5,6,7,8,9]. These products are formed through the non-enzymatic glycation of biomolecules, such as proteins, lipids, and nucleic acids, with an alternative pathway involving the reaction with α-dicarbonyl compounds, which are highly reactive and able to establish covalent bonds [3,10,11]. Upon binding to the receptor for AGEs (RAGE), a cascade of cellular oxidative stress and inflammatory pathways is triggered [12]. AGEs possess the ability to create durable chemical bonds with nearby proteins, especially through cross-linking, altering their structure and function in a process that bypasses enzymatic catalysis [11]. This increase in cross-links, induced by AGEs, leads to a reduction in tissue elasticity and flexibility, thus contributing to the pathogenesis of atherosclerosis, elevated vascular resistance, and CV events (CVEs) [13,14].

In recent years, the emphasis on non-invasive assessment techniques has grown, with skin autofluorescence (SAF) emerging as a pivotal tool. Utilizing the fluorescent properties of certain AGEs, SAF has been recognized as a reliable method for quantifying AGE levels in both diabetic and non-diabetic populations. This approach has gained attention for its ability to deliver precise and reproducible results without necessitating invasive procedures [15,16,17,18]. SAF has been identified as a practical, straightforward method facilitating DM screening in populations without a prior DM diagnosis [18], and has been shown to refine the FINDRISC model, enhancing DM detection strategies [19]. Moreover, its predictive capacity for CV disease (CVD) and mortality has been documented, positioning SAF as an important tool in expanding the understanding of DM screening and CV risk assessment [20,21,22].

The link between SAF and CV mortality in individuals with CKD, DM, or peripheral artery disease (PAD) further emphasizes AGEs’ contribution to vascular complications in CVD through inflammatory mechanisms. Compared with established CV measures, like pulse wave velocity and intima-media thickness, SAF’s simplicity and effectiveness offer a comparative advantage, facilitating vascular health screenings [23,24]. Moreover, SAF’s ability to detect subclinical atherosclerosis, independent of conventional CV risk factors, underscores its significance in identifying early signs of arterial damage, as illustrated in studies by Pan et al. [25]. The association of SAF with adverse CVEs in heart failure (HF) patients also underscores its role in risk stratification [26]. Although reference values for SAF exist for Caucasian populations, significant differences have been noted between various European groups [27]. Calls for more detailed clinical analysis and the creation of tailored reference data for specific demographics have been made since 2014, aiming to improve the test’s utility and accuracy for a wide range of populations [28]. However, it is significant to note that, as of now, no study has specifically assessed the utility of SAF across a broader Eastern European context or the Romanian population.

This study aimed to evaluate the associations between SAF levels, CV risk, and DM chronic complications, within a large cohort of patients with T2DM.

## 2. Materials and Methods

### 2.1. Study Design, Protocol, and Patients

In a single-center, cross-sectional, non-interventional, and consecutive case population-based enrollment study, a total of 896 patients with T2DM were included. All participants were recruited during their regularly scheduled clinical visits between January and July 2019 at Consultmed Hospital in Iasi, Romania.

The inclusion criteria were as follows: patients aged 18 years or older with a confirmed diagnosis of T2DM who had received standard stable management for associated conditions for a minimum of three months, as per the medical guidelines in effect at that time.

Exclusion criteria for this study included patient refusal, individuals under 18 years of age, patients diagnosed with other forms of DM (secondary, type 1, latent autoimmune), those with mental illness, pregnant or breastfeeding women, and patients with an identified presence of autoimmune markers (GADA, ICA, IA-2A, and ZnT8-Ab antibodies).

Utilizing statistical information from the Iasi County population, which was reported as 944,074 inhabitants, with a margin of error of 3.29% and a confidence level of 95%, we calculated a representative sample consisting of at least 885 patients. The remaining patients were included in the analysis.

### 2.2. Demographic, Anthropometric, Clinical, and Laboratory Data Collection

This study involved an analysis of hospitalized patients’ medical records, concentrating on sociodemographic and anthropometric characteristics, comorbidities, laboratory results, DM complications, and treatment plans. The data collected included variables such as residency, age, sex, weight, height, body mass index (BMI), high blood pressure (HBP), atherosclerotic CVD (ASCVD), dyslipidemia, HbA1c levels, estimated glomerular filtration rate (eGFR), uric acid, total cholesterol (total-C) and its fractions, SAF levels, DM complications, and prescribed medications.

### 2.3. Cardiovascular Risk Profile Assessment

The CV risk profile of the patients was assessed according to the ESC/EAS 2019 classification, which was applicable at the time of this study [29]. These guidelines divide patients into moderate-, high-, and very-high-risk categories according to their CV risk levels. Considering that all participants had T2DM, the low-risk category was deemed irrelevant, resulting in their classification into at least the moderate-risk group by default.

Patients under 50 years old with a T2DM duration of fewer than 10 years and no additional risk factors were categorized as moderate risk. The high-risk category included patients who had been living with T2DM for at least 10 years but showed no signs of target organ damage (TOD) (microalbuminuria, retinopathy, or neuropathy). This category also encompassed patients with significantly elevated individual risk factors, as specified by the ESC/EAS guidelines. Those with an eGFR between 30 and 59 mL/min/1.73 m^2^ were classified as high risk as well [29].

Within the very-high-risk group, individuals meeting the following criteria were considered: T2DM diagnosis accompanied by TOD or those with at least three major risk factors. Additionally, this group included individuals with established ASCVD, as determined by the ESC/EAS guidelines. Patients with severe CKD, marked by an eGFR lower than 30 mL/min/1.73 m^2^, were also placed in the very-high-risk category [29].

### 2.4. Measurements of SAF Levels

The assessment of SAF was performed non-invasively by placing intact skin on the ventral side of the forearm, following the manufacturer’s instructions provided with the AGE Reader™, developed by DiagnOptics Technologies in Groningen, The Netherlands. This device measures the accumulation of AGEs within approximately 1 mm beneath the epidermis by utilizing the autofluorescent properties of specific AGEs. It features a 4 cm^2^ aperture that allows light emission through a UV-A lamp. Upon contact, this emitted light reflects off the epidermis before being detected by a spectrometer within a wavelength range of 300 to 600 nanometers and is processed using specialized software. The results are then presented in arbitrary units (AUs). This methodology has undergone rigorous testing with additional technical specifications detailed in previous publications [15,17,18].

### 2.5. Diabetes Complication Assessment

T2DM complications, such as nephropathy, microalbuminuria, retinopathy, and diabetic sensory peripheral neuropathy (DSPN), were identified and included in this study.

The diagnosis of DSPN was confirmed either by a previously documented diagnosis of DSPN or by clinical examination incorporating at least two of the following tests: evaluation of thermal or pinprick sensibility (assessing small-fiber function) and assessment of vibration sensing using a 128 Hz tuning fork (evaluating large-fiber function) and the 10 g monofilament test. Cardiac autonomic neuropathy (CAN) was evaluated with Sudoscan^®^. The Sudoscan^®^ device, developed by Impeto Medical in Paris, France, and FDA approved, offers a non-invasive method for assessing sudomotor function. It measures the electrochemical skin conductance (ESC) of sweat glands using electrodes for the hands and feet. The device assesses the release of chloride ions by sweat glands under low voltage, conducting a brief assessment that lasts approximately 2 min and does not require any special conditions or preparations. Sudoscan’s algorithms combine ESC with demographic and health data to generate scores for CAN Risk (CANRS) and kidney dysfunction risk, providing insights into the risks of CAN and CKD [30,31,32]. The CAN score and nephropathy (NEPHRO) score were reported for each patient.

Identification of diabetic retinopathy (DR) was achieved either through a documented previous diagnosis in the patient’s medical record or via an ophthalmologic examination conducted at our medical center, which included a fundoscopic evaluation.

CKD was defined as either a pre-existing diagnosis or persistent evidence of a urine albumin/creatinine ratio (UACR) equal to or greater than 30 mg/g, and/or a consistently low eGFR below 60 mL/min/1.73 m^2^, and/or the presence of structural abnormalities or hematuria, as outlined in the guidelines [33].

### 2.6. Statistical Analysis

The information was structured into Excel databases and subjected to analysis using both the Excel program for Windows 11 and Gnu PSPP 1.4.1 software, enabling a thorough statistical assessment of the gathered parameters. Results are presented as mean (standard deviation—SD) or median (interquartile range—IQR). The distribution of data was tested using the Kolmogorov–Smirnov test. In univariate correlation analysis, Pearson or Spearman coefficients were selected depending on variables’ distribution. First, a receiver operating curve (ROC) was used to identify the cut-off for SAF determination. The cut-off value was determined using Youden’s method as the value in which the sum of sensitivity (Sn) and specificity (Spe) is maximized. Second, using logistic regression, we estimated the probability of the binary outcome based on the SAF cut-off. Additional multivariate regression models for predicting SAF as a continuous variable were generated. A *p*-value less than 0.05 was considered statistically significant.

### 2.7. Ethical Aspects of the Research

The investigation adhered to the principles outlined in the Declaration of Helsinki and received approval from the Institutional Ethics Committee of Consultmed Hospital in Iași County, Romania (protocol number CMD102018006, dated 18 October 2018). The objectives and methods of this study were explained to the patients, who consented to the utilization and publication of the data collected, and informed consent was obtained.

## 3. Results

**Figure 1:**
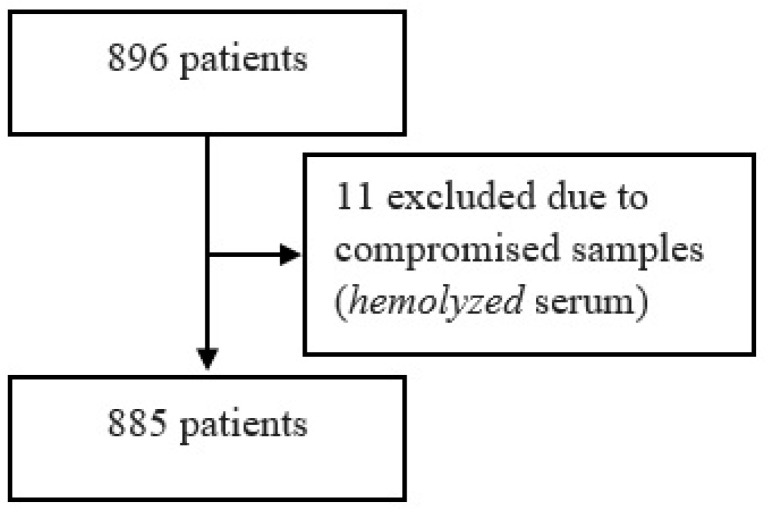
Patients inclusion flowchart.

During their regularly scheduled appointments at the Consultmed Hospital, the initial cohort of 1000 patients who met the inclusion criteria were invited to participate in this study. During the enrolment visit, medical records were collected without any subsequent study-related appointments being scheduled. Among the 896 patients who provided informed consent, 11 were excluded due to extreme laboratory results indicating compromised blood samples or errors in laboratory analysis (Figure 1).

Our study cohort demonstrated a relatively balanced distribution, consisting of 53.7% females and 46.3% males, with no statistically significant differences between genders. The mean age of the analyzed patient sample was 62.9 ± 7.7 years, with 74.69% residing in urban areas. Anthropometric characteristics included a mean BMI of 32.3 ± 5.3 kg/m^2^, a mean waist circumference of 104.74 ± 11.7 cm, a mean hip circumference of 109.34 ± 9.94 cm, and a mean waist–hip ratio of 0.96 ± 0.074, as detailed in Table 1.

Table 2 outlines the patient’s risk factors. The prevalence of obesity stands at 64.6%, HBP at 83%, with a mean systolic blood pressure (BP) of 132 ± 16.2 mm Hg and a mean diastolic BP of 80 ± 9.6 mm Hg; 13.9% of patients present with CVD. The lipid profile includes total-C at 185.1 ± 43.3 mg/dL, triglycerides at 142 (93) mg/dL, low-density lipoprotein cholesterol at 107.73 ± 36.01 mg/dL, and high-density lipoprotein cholesterol at 44.9 ± 11.8 mg/dL; the mean eGFR is 87.5 ± 20.6 mL/min/1.73 m^2^. The distribution of patients across CV risk categories is as follows: 6.1% are classified in the moderate-risk category, 1.13% in the high-risk category, and 92.77% in the very-high-risk category.

Table 3 provides a summary of the DM characteristics among the patients, indicating an average DM duration of 9.0 ± 4.4 years, with no significant differences observed across genders; the mean HbA1c level is 7.1% ± 1.3. Current DM complications include DSPN at 67.9%, DR at 4.29%, CKD at 8.7%, albuminuria at 6.21%, and ASCVD at 13.9%.

The use of pharmacological therapies in the analyzed cohort is presented in Table 4.

**Figure 2:**
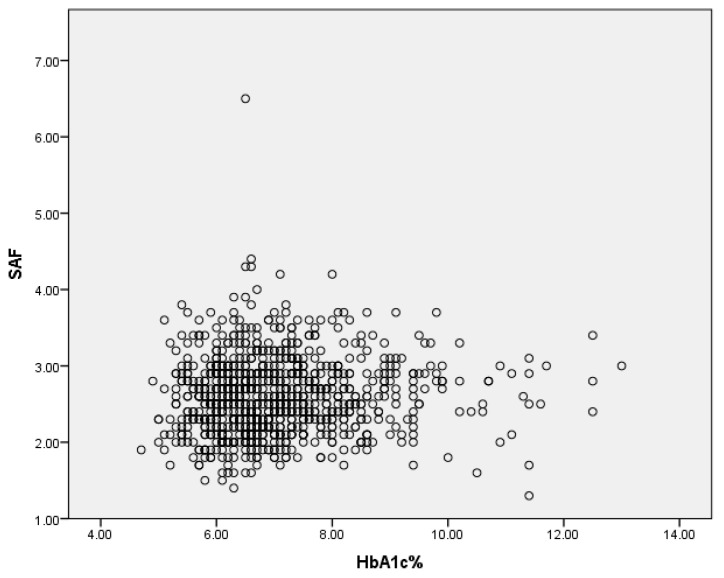
Correlation between SAF and HbA1c.

In univariate analysis, SAF was significantly correlated with age (Pearson coefficient = 0.294, *p* < 0.001), CAN score (Pearson coefficient = 0.136, *p* < 0.001), and NEPHRO score (Pearson coefficient = −0.230, *p* < 0.001). There was a small significant correlation between SAF and HbA1c (Spearman’s rho = 0.091, *p* = 0.007, Figure 2).

Additionally, after adjusting for age and eGFR in the multivariate regression model, HbA1c values were found to correlate with SAF levels. Specifically, for each increase of 1 standard deviation (SD) in HbA1c value, an observed increase of 0.105 SDs in SAF levels was noted (Nagelkerke R^2^ = 0.110; *p* < 0.001).

**Figure 3:**
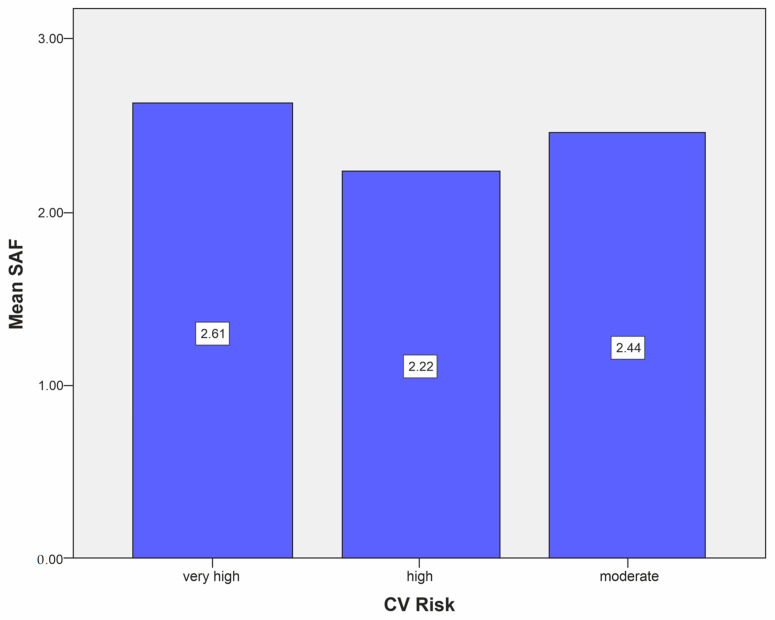
Mean SAF level depending on CV risk category.

We compared the mean SAF levels in T2DM patients across different HbA1c targets. The mean SAF level was significantly higher in subjects with an HbA1c > 7% (2.65 ± 0.53) compared to those with an HbA1c < 7% (2.56 ± 0.53), *p* = 0.018. Additionally, SAF levels and DM duration were found to be independent, r = −0.028, *p* = 0.401. Regarding CV risk categories, patients in the very-high-risk CV group exhibited significantly higher SAF levels (2.61) compared to those in the high-risk (2.22) and moderate-risk (2.44) groups, respectively, *p* = 0.003, as illustrated in Figure 3.

When we compared SAF levels in different DM complications groups—DSPN, DR, CKD, and albuminuria—we did not observe any significant differences, except for CKD, as seen in Table 5.

**Figure 4:**
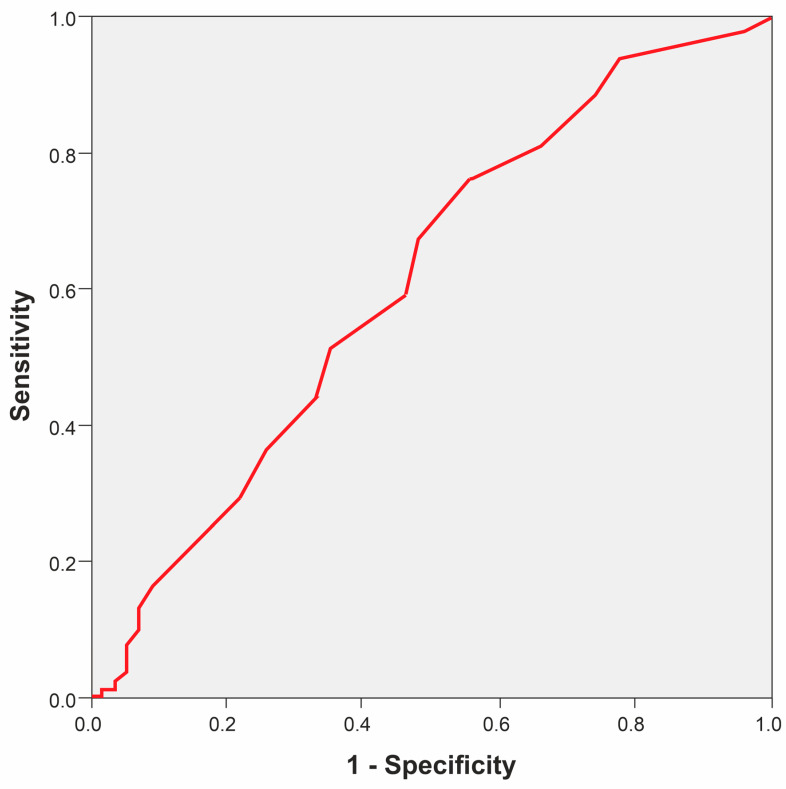
ROC curve for SAF levels in predicting very high CV risk.

When evaluating the cut-off value for SAF levels in predicting very high CV risk (2.35), an Sn of 67.7% and an Spe of 56.2% were achieved. The AUC value was 0.634 (95% CI: 0.560–0.709), with a *p*-value of 0.001, as depicted in Figure 4.

In the subgroup analysis, only age and HbA1c level showed significant differences in comparison to the 2.35 SAF cut-off level, with a *p*-value of 0.001. No significant differences were observed in terms of gender, DM duration, and DM complications (Table 6).

Logistic regression models (Table 7) for predictors of SAF > 2.35 have demonstrated that elevated SAF levels are significantly associated with very high CV risk, especially after adjusting for age, gender, and HbA1c level. Age was notably associated with an increased risk (OR: 1.072; 95% CI: 1.048–1.096; *p* = 0.001), indicating that older patients have a greater probability of being included in the very high CV risk group, with elevated SAF levels. Additionally, gender was identified as a significant factor (OR: 1.426; CI: 1.057–1.923; *p* = 0.02), as well as higher HbA1c levels, which further amplified this risk (OR: 1.171; CI: 1.039–1.321; *p* = 0.01).

## 4. Discussion

Our research assessed SAF in a large cohort of T2DM patients and examined the cross-sectional relationships between SAF and microvascular complications of DM. We also sought to identify associations between AGEs and CV risk categories and propose a cut-off value for SAF levels in predicting very high CV risk.

The findings indicate that, within this study’s population, the SAF level potentially acts as a non-invasive indicator for detecting high CV risk among individuals with T2DM. In our cohort, significant associations were found between SAF levels and various parameters: such as age, CAN score reflecting CAN, NEPHRO score signaling kidney dysfunction risk, and glycemic control as measured by HbA1c levels. However, it is important to note that when we compared SAF levels across various DM complication groups—including DSPN, DR, CKD, and albuminuria—no significant differences were noted, with the exception of CKD. These findings underscore the importance of a multifactorial and individualized approach to risk assessment in patients with T2DM, integrating measures of glycemic control, as well as indicators of metabolic stress, such as SAF. In a multivariate regression analysis, accounting for factors, such as age and eGFR, HbA1c values were identified as independent predictors of SAF levels. Specifically, for each SD increase in HbA1c values, there was an observed rise of 0.105 SD in SAF levels. This indicates that as HbA1c levels (a measure of glycaemic control over the past 2 to 3 months) increase, so do SAF levels, suggesting a greater accumulation of AGEs, as assessed by SAF. This underscores the connection between inadequate glycaemic control and AGE accumulation, which contributes to the risk of complications in managing DM. Additionally, we assessed SAF’s effectiveness as a screening tool, determining the optimal SAF threshold that balances Sn and Spe, along with the area under the ROC curve (AUC) as a measure of SAF’s diagnostic accuracy.

### 4.1. SAF and Microvascular Complications of DM (Neuropathy, Retinopathy, Nephropathy)

Although no significant differences in SAF levels were observed among patients with various DM complications (DSPN, DR, albuminuria) within our study’s population, an exception was noted for CKD. Patients with CKD (eGFR below 60 mL/min/1.73 m^2^) exhibited higher SAF levels (*p* < 0.003), suggesting a potential association between elevated skin AGEs and CKD presence. This could imply that SAF as a marker of AGE accumulation might have a direct correlation with CKD in our cohort, in contrast to the other chronic complications included in the analysis. While our study indicates that SAF’s effectiveness as a screening tool for microvascular complications, including DSPN, DR, and albuminuria, may be constrained within our study’s population, it also underscores the potential for SAF’s broader applicability and predictive value for other complications. This highlights the necessity for further research involving a more varied group of individuals and the importance of longitudinally assessing our study’s population to fully explore SAF’s utility.

Contrary to our findings, Hosseini et al., in their recent systematic review and meta-analysis of 29 studies—25 of which were cross-sectional, with 13 conducted within European cohorts—identified a pronounced link between SAF levels and suboptimal DM management, as reflected by the last HbA1c (0.21 with a 95% CI of 0.13–0.28), and a heightened risk for DR, DSPN, nephropathy, and macrovascular complications. This analysis highlighted heterogeneity among the reviewed studies, emphasizing the necessity for cautious interpretation of these findings due to the variations in study designs, methodologies, and the populations studied. Despite these variations, a consistent statistical significance was observed (*p* < 0.05), confirming SAF’s relevance as a non-invasive marker for both micro- and macrovascular complications associated with DM. This insight into SAF’s predictive capacity for the early detection of irreversible DM complications reflects its potential utility in further research with larger and more diverse cohorts alongside extended follow-up. So, it is essential to fully ascertain SAF’s role in the clinical evaluation of DM complications, as underscored by Hosseini’s analysis [34].

The connection between SAF and the complexity and severity of DM complications was also explored in a cross-sectional analysis of 825 patients with T2DM, revealing a significant correlation between skin AGEs and DR, diabetic kidney disease, CVD, and DSPN, noting that SAF levels increased with the progression of complications. Furthermore, the study identified distinct associations between SAF and demographic factors, including age, gender, and stimulated C-peptide, as well as clinical indicators like creatinine and fatty liver. The authors proposed an AGE-based risk score for diabetic complications, capable of predicting the likelihood of T2DM complications, thereby underscoring the significant predictive role of SAF [35].

As DSPN and its subtypes represent a prevalent complication of DM, SAF has been explored as a potential screening method for various forms of neuropathy in T2DM patients. In a study including 132 participants, the authors concluded that skin AGEs could serve as a screening tool for DSPN and CAN in T2DM patients, noting their potential utility in clinical settings. However, the study acknowledged the method’s moderate to low Spe, suggesting that additional diagnostic procedures might be necessary for confirmation if screening results are positive, to enhance its accuracy. Compared to our cohort, T2DM patients in the Papachristou et al. study were slightly older (64.57 ± 8.21 years versus 62.9 ± 7.7 years) and had a longer history of hyperglycemia, as indicated by their higher DM duration (14.5 years [range 7.00–20.00] versus 9.0 ± 4.4 years). The optimal cut-off for overall DSPN was determined to be SAF ≥ 2.95, which is higher than our mean SAF score across the entire cohort (2.6 ± 0.5) [36]. In a recent longitudinal study, SAF levels were shown to predict foot ulcers in a cohort of 517 patients with a mean HbA1c of 8.7 ± 1.8%, an average age of 62 ± 9 years, and an average DM duration of 14 ± 10 years [37]. The population studied in our research was notably different: our study included a cohort of T2DM patients who had a lower average HbA1c level and fewer patients diagnosed with chronic DM complications (DR, 4.29% versus 26.7%; CKD, 8.7% versus 44.9%; and macroangiopathies, 13.9% versus 33.8%). In a multicenter study involving 497 patients where neuropathy was assessed using three validated tools, the Toronto Clinical Neuropathy Score, the Neuropathy Disability Score, and the Neuropathy Symptoms Score, SAF levels were found to be not only elevated but also progressively increased with the severity of DSPN. These levels were associated with both the presence of symptoms and nerve deficits and demonstrated correlations with DM duration, glycemic control, and serum creatinine levels [38]. The various pathways leading to AGE formation—glycolytic dysfunction, lipid peroxidation, and glucotoxicity—play significant roles in the development of diabetic neuropathy, with varied impacts on its pathogenesis, including alterations in heart rate variability and changes in vibration perception thresholds, as underlined by Al-Saoudi et al. [39].

Increased levels of AGEs have been correlated with DR, as evidenced by multiple studies [40,41,42,43,44,45]. Yasuda and colleagues noted a correlation between skin AGE levels and the severity of DR in T2DM patients. In their examination of 67 T2DM patients and age-matched controls, significant increases in skin AGE levels were observed alongside DR progression. Furthermore, logistic regression analysis identified skin AGE levels as an independent predictor of proliferative DR (PDR) with an odds ratio (OR) of 17.2 (*p* < 0.05). The authors proposed measuring skin AGE levels as a useful tool for assessing DR risk and as a surrogate marker for non-invasively evaluating DR progression and severity in T2DM patients [42]. Hirano et al. reported similar findings in a population of 132 T2DM patients and non-diabetic controls, with a mean age of 63.7 ± 12.2 years, a HbA1c level of 7.5 ± 1.7%, and a DM duration of 13.2 ± 9.9 years. They found SAF to be correlated with DR severity but not with the prevalence or severity of diabetic macular edema (DME). This study also identified SAF as an independent factor indicating the occurrence of PDR and suggested that SAF can predict the risk of severe DR. The proportion of patients with DM complications was also higher than in their cohort, with only 26.1% showing no apparent DR [43]. A strong association between the presence of PDR and SAF, as well as its potential as a predictor for DR severity, was also reported by Takayanagi et al. Their study included T2DM patients with a mean age of 68.4 ± 13.7 years and a higher mean HbA1c [44].

A recent systematic review and meta-analysis, which included the majority of studies conducted in Caucasian populations, assessed the accuracy of SAF in the early detection of DR. This is particularly important because devices used for SAF readings have lower performance in highly pigmented skin. The review concluded that SAF demonstrates adequate accuracy for use in clinical settings for DR screening in patients with T2DM. The diagnostic OR (dOR) was 5.11 (*p* < 0.001), indicating a statistically significant association. However, there was notable heterogeneity in Spe but not in Sn. The authors concluded that while SAF is unlikely to replace ocular fundus inspection, it may serve as a convenient screening technique, especially in situations with limited resources. Further research is necessary to determine the reference values and the consistent usefulness of this tool across various populations [45]. Błaszkiewicz et al. describe the pathophysiological impact of AGEs on DR, noting their role in compromising neurovascular integrity through oxidative stress and inflammation The binding of AGEs to RAGE on retinal cells initiates harmful signaling pathways, especially in Müller cells. Once activated, these cells increase VEGF production and inflammatory responses, leading to neovascularization and retinal damage. Moreover, AGEs cause pathological alterations, including protein cross-linking and endoplasmic reticulum (ER) stress, worsening retinal cell dysfunction and blood–retinal barrier degradation. The authors underscore SAF’s role in early DR detection and agree with previous studies on its efficacy as a non-invasive marker, suggesting that non-invasively measuring AGE accumulation offers a more accurate reflection of AGE levels than analyzing serum concentrations, which may not accurately represent tissue AGE levels and varies with the molecules’ half-life [46].

In discussing renal complications in DM, AGEs are linked to the onset and progression of CKD. This process is initiated through AGEs’ interaction with their receptor, triggering reactions that activate the inflammatory oxidative stress axis previously described, thereby influencing the progression of complications. Additionally, the AGE-RAGE interaction accelerates atherosclerosis, supports alterations in myocardial structure with cardiomyocyte impairment, leading to HF progression, and raises the risk for CVE, the main cause of death in patients living with both conditions, CKD and DM. Higher AGE levels in this population also contribute to muscle wasting and nutritional imbalances, accelerating the decline in the overall health status of this vulnerable patient group [47]. Fraser et al. investigated whether SAF could be a marker of all-cause mortality risk in stage 3 CKD. Initially, those in the highest quartile for skin AGEs faced a greater risk of all-cause mortality compared to patients with lower SAF levels. However, this association diminished after adjusting for confounding factors, such as CVD, glucose levels, BMI, albuminuria, and renal function. SAF in CKD reflects the accumulation of AGEs and cumulative metabolic stress, indicating the need for further research to understand SAF’s predictive role in these patients [48]. On the contrary, Rigalleau et al. and Jin et al. reported that SAF is independently associated with renal outcomes in T2DM patients. Both studies affirm the significance of SAF; the former underscores its value as an independent prognostic marker for kidney dysfunction, separate from traditional renal markers, while the latter confirms its importance concerning macroangiopathy [49,50]. From these data, it seems evident that there exists a robust and firmly established correlation between SAF and DR, likely attributable to the direct influence of AGEs on retinal microvessels [45,51]. However, the connection between SAF and neuropathy or nephropathy is less pronounced, and SAF’s role and predictive values may differ across various complications, potentially due to other pathophysiological pathways beyond AGE accumulation. The increased levels of AGEs in patients with DM also indicate the metabolic burden represented by elevated glucose levels, as well as atherogenic dyslipidemia, high levels of pro-inflammatory cytokines, and disruptions in reactive oxygen species homeostasis [34,52,53]. A systematic review and meta-analysis from 2023, evaluating 33 case–control studies mostly conducted in Europe, compared AGE levels in individuals with and without DM to assess SAF’s validity in DM populations. Despite significant associations between SAF, a higher BMI, AGE accumulation, and DM complications, the authors conclude that more research is necessary to fully grasp SAF’s usefulness as a surrogate biomarker in DM due to study variability. Factors like BMI, gender, age, and metabolic load significantly impact SAF, suggesting its potential utility as an indicator for MetS or DM [54].

Skin levels of AGEs, as determined by biopsy specimens, were linked to the onset and advancement of DM complications in both type 1 and T2DM in the DCCT-EDIC and UKPDS cohorts, even after adjusting for HbA1c levels [55,56]. High concentrations of AGEs in skin collagen, also determined through skin biopsies, were found to forecast the quick progression of microvascular complications in DM over a span of six years in T2DM patients, indicating the need for stricter DM control [57]. Still, evaluating AGEs through skin biopsy is impractical for routine clinical use [56].

Our study findings might be attributed to differences in demographic characteristics such as age, ethnicity, and DM durations. For instance, the rate of AGE accumulation and tissue impact is known to vary with age and a patient’s metabolic control over time, which are specific to each study’s population characteristics [58]. Additionally, DM duration is also likely to explain the differences in our findings, as a longer DM duration is generally linked to higher AGE accumulation, influencing relationships between SAF levels and complications in the microvasculature [56]. The relatively low prevalence of microvascular complications in our study population compared to other cohorts may have played a critical role in the observed lack of significant association between SAF levels and these complications. In populations where DM-related complications are less common, the statistical power to detect associations between SAF levels and such complications is diminished, which could result in a failure to observe significant associations, even if the pathophysiological link exists.

### 4.2. SAF and CV Risk in DM

AGEs are known to play a significant role in promoting CVD and mortality by influencing the structure and function of vascular and myocardial tissues. They cause stiffness in the vascular walls by altering the physical properties of extracellular matrix proteins and contribute to impaired vasodilation and reduced vascular flexibility by affecting endothelin-1 production and reducing nitric oxide levels. Additionally, AGEs, through their interaction with RAGEs, lead to a range of detrimental changes, including atherosclerosis, thrombosis, and further vascular constriction. RAGEs also facilitate fibrosis by increasing TGF-β levels [59] and altering calcium metabolism within the myocardium [60,61].

The vast majority of our patients (821, 92.77%) were in the very-high-risk category as per the ESC/EAS 2019 risk stratification applicable at the time of the cross-sectional evaluation of this cohort. Patients included in the very-high-risk category showed the highest average mean SAF levels. This finding, especially with a statistically significant difference (2.61AU; 2.22AU vs. 2.44AU; *p* = 0.003) suggests that higher SAF levels are associated with increased CV risk. This is consistent with previous research and suggests that AGEs, assessed by SAF, contribute to CVD progression in T2DM individuals. For the very high CV risk category, an SAF value greater than 2.35 was found to be a good predictor, having an Sn of 67.7% and an Spe of 56.2% (AUC = 0.634; 95% CI 0.560–0.709; *p* = 0.001). In a retrospective cohort study of 504 individuals followed for an average duration of 54 months, it was revealed that 14% of them experienced a CVE. The levels of SAF were significantly higher in patients who experienced a CVE compared to those who did not (2.89 vs. 2.63 AU, *p* = 0.002). This association between elevated SAF levels and the occurrence of CVEs remained statistically significant even after adjusting for other variables, including glycemic control, HBP, lipid profile abnormalities, other vascular complications, and DM treatments. Patients with higher SAF levels had a lower rate of CVE-free survival. Furthermore, even among those without macroangiopathy at baseline, individuals with higher SAF levels had a lower CVE-free survival rate compared to those with lower SAF levels. This suggests that SAF may serve as a useful marker for identifying patients at increased risk of CV complications [62].

A study incorporating 2349 participants from the Lifelines Cohort Study—all with T2DM, either newly diagnosed or already diagnosed, but without clinical CVD—investigated the predictive value of elevated SAF levels for the onset of CVD and mortality. Over an average follow-up of 3.7 years, the findings revealed that individuals with higher SAF levels had a substantially greater risk of experiencing CVEs or mortality. This correlation persisted even after adjusting for traditional risk factors, such as BP and cholesterol levels, indicating that SAF is a significant and independent predictor of new CVEs and mortality among T2DM patients (OR 2.59, 95% CI 2.10–3.20, *p* < 0.001). Notably, “new” T2DM cases exhibited lower SAF values than those with an established diagnosis, suggesting that prolonged hyperglycemia contributes to higher SAF levels in the latter group. In this study, SAF was a more potent predictor of CV complications and mortality in people with T2DM compared to traditional indicators [63].

Refining the identification in the T2DM populations of those patients with a higher risk for CVEs, and, hence, suitable candidates for revascularization procedures, has been investigated in a recent study by Alkhami et al. SAF levels were assessed at baseline in 477 T2DM patients, and new revascularizations (coronary and lower-limb arteries) were documented throughout a 54-month period. The study reported that patients with SAF levels greater than 2.6 AU, which was the median value for the study population, had a significantly higher incidence of revascularizations compared to individuals with lower SAF values, even after accounting for confounding factors. SAF offers the potential to improve the screening process for advanced investigations and possible revascularizations, resulting in better risk classification in T2DM patients [64]. The relationship between SAF and subclinical CVD was particularly strong in DM patients, emphasizing the importance of better understanding CV risk in T2DM populations, as shown in the Rotterdam study [65]. In our cohort, the optimum cut-off point for SAF levels in predicting a very high CV risk was 2.35 AU, which was associated with augmented HbA1c levels and older age.

### 4.3. Strengths and Limitations

Our results are restricted to a single-center cohort, necessitating caution when generalizing the results. Additionally, our group had a small sample size of participants with chronic DM microvascular complications, and the statistical power of our inference is restricted. As per the cross-sectional design of our study, this analysis provides a snapshot of a single point in time, describing the current state of a representative sample in Romanian T2DM patients, and it has the limitation that it cannot infer temporal sequences or causality from these associations described above. Nevertheless, the cohort examined was large and well characterized, and it remains a feasible scenario in our resource-constrained setting, as it provides a comprehensive overview of DM complications and the distribution of CV risk categories in correlation with SAF levels. One notable strength of our study is the cohort’s well-controlled BP values and nearly optimal metabolic control. However, reports indicate that despite meeting glycemic control targets, most patients do not achieve a comprehensive treatment goal encompassing HbA1c, BP, and lipid levels, and there is an underutilization of newer antidiabetic medications with potential cardio- and reno-protective benefits [66,67,68,69]. Our study may constitute the baseline for a longitudinal follow-up of the same cohort. This approach will offer an opportunity for the development of targeted interventions to address the unmet needs of the Romanian T2DM population and enhance our understanding of disease progression and management over time.

In our research, we assessed the accumulation of AGEs using SAF. There are both advantages and drawbacks to this method. In a previous study, Lutgers et al. highlighted that autofluorescence readers have limitations, as non-fluorescent AGEs will not be measured by the method, and substances with auto-fluorescent properties may introduce confounding factors [70]. Several limitations have been identified in SAF measurements, including the fact that they are not suitable for individuals with high levels of skin pigmentation, and that various substances, such as creams, sunscreens, or extreme blood flow variations, may affect the accuracy of the measurements [71]. Despite this, evaluating skin AGEs by SAF has shown a stronger and more robust correlation with CVD risk than circulating AGE levels. Serum AGEs have had an unclear correlation with CVD due to their short activity duration and alterations influenced by various dietary habits and metabolic factors [72].

A noted constraint of our study is the incomplete available data regarding specific risk factors, such as smoking. Future studies should include smoking as a variable to provide a more comprehensive understanding of its effects on SAF’s predictive accuracy for CV complications.

As far as we are aware, this research marks the first exploration into establishing a threshold for SAF’s correlation with CV risk and DM complications among Romanian individuals with T2DM. We aim to extend this research longitudinally and expand the range of participants. This initiative is directed toward validating the reliability of SAF measurement as a tool for estimating CVD risk specifically within our demographics. By offering a non-invasive and accessible method, SAF holds the potential to significantly assist clinicians in identifying patients at high CV risk and making informed decisions about treatment adjustments, highlighting its critical clinical significance. Further research could explore how SAF, in conjunction with other biomarkers and clinical assessments, can be integrated into comprehensive risk assessment models to improve patient care and outcomes in DM management.

## 5. Conclusions

Increased CV risk and higher HbA1c values are associated with increased SAF levels in patients with T2DM. The optimal threshold of SAF levels in predicting very high CV risk in patients with T2DM is 2.35. The presence of CKD in patients with T2DM was associated with higher SAF levels, while the presence of other T2DM degenerative chronic complications was not significantly associated with SAF levels. SAF levels above the obtained threshold were associated with higher HbA1c and higher patient age. 

## Figures and Tables

**Table 1:** Patients’ characteristics.

Characteristic	*n* = 885
Demographics	
Urban, %, *n*	74.69% (661)
Age (years), mean (SD)	62.9 ± 7.7
Women, %, (*n*)	53.7% (475)
Anthropometrics	
BMI (kg/m^2^), mean (SD)	32.3 ± 5.3
Waist circumference (cm), mean (SD)	104.74 ± 11.7
Hip circumference (cm), mean (SD)	109.34 ± 9.94
Waist–hip ratio, mean (SD)	0.96 ± 0.074

SD—standard deviation; BMI—body mass index.

**Table 2:** Patients’ risk factors.

Characteristic	*n* = 885
Risk factors	
Mean SAF level, mean (SD)	2.6 ± 0.5
Mean SAF level in the moderate CV risk category, mean (SD)	2.44 ± 0.55
Mean SAF level in the high CV risk category, mean (SD)	2.22 ± 0.29
Mean SAF level in the very high CV risk category, mean (SD)	2.61 ± 0.5
Obesity, %, (*n*)	64.6% (572)
HBP, %, (*n*)	83% (737)
SBP (mm Hg), mean (SD)	132 ± 16.2
DBP (mm Hg), mean (SD)	80 ± 9.6
CVD, %, (*n*)	13.9% (123)
Total-C (mg/dL), mean (SD)	185.1 ± 43.3
HDL-C (mg/dL), mean (SD)	44.9 ± 11.8
TGs (mg/dL), median (interquartile range)	142 (104 to 197)
LDL-C (mg/dL), mean (SD)	107.7 ± 36.0
Creatinin (mg/dL), median (interquartile range)	0.83 (0.68 to 0.9)
eGFR (mL/min/1.73 m^2^)	87.5 ± 20.6
Moderate CV risk category, %, *n*	6.1% (54)
High CV risk category, %, *n*	1.13% (10)
Very high CV risk category, %, *n*	92.77% (821)

SAF—skin auto fluorescence; SD—standard deviation; HBP—high blood pressure; SBP—systolic blood pressure; DBP—diastolic blood pressure; CVD—cardiovascular disease; total-C—total cholesterol; HDL-C—high-density lipoprotein cholesterol; TGs—triglycerides; LDL-C—low-density lipoprotein cholesterol; eGFR—estimated glomerular filtration rate.

**Table 3:** Diabetes mellitus characteristics.

DM Characteristics	*n* = 885
DM mean duration, mean (SD)	9.0 ± 4.4
HbA1c (%), mean (SD)	7.1 ± 1.3
DSPN, %, *n*	67.9% (601)
DR, %, *n*	4.29% (38)
CKD, %, *n*	8.7% (76)
Stage V CKD, %, *n*	0
Stage IV CKD, %, *n*	3 (0.34%)
Stage IIIa CKD, %, *n*	13 (1.47%)
Stage IIIb CKD, %, *n*	60 (6.78%)
Albuminuria, %, *n*	6.21% (55)

DM—diabetes mellitus; DSPN—diabetic sensory peripheral neuropathy; DR—diabetic retinopathy; CKD—chronic kidney disease.

**Table 4:** Therapies used in the studied cohort.

Characteristic	*n* = 885
Glucose-lowering medication usage	
Insulin, %, (*n*)	25.2% (223)
Metformin, %, (*n*)	87.0% (687)
DPP-4i, %, (*n*)	13.0% (115)
GLP-1 RAs, %, (*n*)	8.1% (71)
SGLT2i, %, (*n*)	3.9% (34)
Sulfonylurea, %, (*n*)	13.1% (116)
Thiazolidinediones, %, (*n*)	1.35% (12)
Other therapies	
ACEi/ARBs, %, (*n*)	61.5% (544)
Calcium channel blockers, %, (*n*)	33.1% (293)
Beta-blockers, %, (*n*)	54.57% (483)
Antiagregant, %, (*n*)	44.85% (397)
Statin, %, (*n*)	67.0% (593)
Ezetimibe, %, (*n*)	4.5% (40)
Fibrate, %, (*n*)	8.7% (77)
Non-vitamin K antagonist oral anticoagulants, %, (*n*)	1.8% (16)
Vitamin K antagonists, %, (*n*)	1.12% (10)

DPP-4i—dipeptidyl peptidase 4 inhibitors; GLP-1 RAs—glucagon-like peptide 1 receptor agonist; SGLT2i—sodium–glucose cotransporter-2 inhibitors; ACEI/ARBs—angiotensin-converting enzyme inhibitors/angiotensin receptor blockers.

**Table 5:** Mean SAF level depending on DM complications.

DM Complications	*n*	Present	Absent	Student’s *t*-Test
DSPN	601	2.61 ± 0.51	2.60 ± 0.49	*p* = 0.792
DR	38	2.58 ± 0.44	2.61 ± 0.51	*p* = 0.746
CKD	76	2.76 ± 0.49	2.58 ± 0.51	*p* = 0.003
Albuminuria	55	2.56 ± 0.50	2.61 ± 0.51	*p* = 0.497

DM—diabetes mellitus; DSPN—diabetic sensory peripheral neuropathy; DR—diabetic retinopathy; CKD—chronic kidney disease.

**Table 6:** Subgroup analysis based on SAF cut-off.

Parameters	SAF ≤ 2.35 (*n* = 301)	SAF > 2.35 (*n* = 584)	Statistic Tests	*p*
Age, mean ± SD	60.06 ± 8.42	64.30 ± 6.86	Student’s *t*-test	<0.001
Female, *n*, (%)	163 (54.2%)	312 (53.4%)	Chi2 test	0.837
Age of DM, mean ± SD	8.78 ± 4.68	9.06 ± 4.25	Student’s *t*-test	0.369
HbA1c mean ± SD	6.86 ± 1.12	7.17 ± 1.30	Student’s *t*-test	<0.001
DSPN, *n*, (%)	200 (66.44%)	401 (68.66%)	Chi2 test	0.733
DR, *n*, (%)	13 (4.31%)	25 (4.28%)	Chi2 test	0.937
CKD, *n*, (%)	5 (1.66%)	12 (2.05%)	Chi2 test	0.711
Albuminuria, *n*, (%)	19 (6.31%)	36 (6.16%)	Chi2 test	0.880

SAF—skin autofluorescence; SD—standard deviation; DM—diabetes mellitus; DSPN—diabetic sensory peripheral neuropathy; DR—diabetic retinopathy; CKD—chronic kidney disease.

**Table 7:** Logistic regression models for predictors of SAF > 2.35. Independent variables: age, female gender, HbA1c, DSPN, DR, CKD, albuminuria.

Logistic Regression Models (Very High Risk—Yes/No)	Predictors	OR (95% CI)	*p*
Model adjust 1	Age	1.071 (1.049–1.094)	0.001
Model adjust 2	Age	1.067 (1.044–1.091)	0.001
	Female gender	1.408 (1.046–1.895)	0.024
Model adjust 3	Age	1.072 (1.048–1.096)	0.001
	Female gender	1.430 (1.061–1.928)	0.019
	HbA1c	1.322 (1.149–1.522)	0.010

OR—odds ratio; CI—confidence interval.

## Data Availability

The archived dataset analyzed during the study is available upon request.
